# Supplementary material for: TPP-Based Nanovesicles Kill MDR Neuroblastoma Cells and Induce Moderate ROS Increase, While Exerting Low Toxicity Towards Primary Cell Cultures: An In Vitro Study
Source: Int J Mol Sci. 2025 May 22;26(11):4991. doi: 10.3390/ijms26114991 (PMC12154426; doi:10.3390/ijms26114991)
Supplement: Supplementary file 1 [file ijms-26-04991-s001.zip › ijms-3599142-supplementary.pdf]

Supplementary Materials

TPP-Based Nanovesicles Kill MDR Neuroblastoma Cells and Induce Moderate ROS Increase, While Exerting Low Toxicity Towards Primary Cell Cultures: An In Vitro Study

Silvana Alfei [1,*], Carola Torazza [1], Francesca Bacchetti [1], Marco Milanese [1,2], Mario Passalacqua [3,4], Elaheh Khaledizadeh [3], Stefania Vernazza [3,4], Cinzia Domenicotti [2,3,4,*] and Barbara Marengo [2,3,4]

[1] Department of Pharmacy, University of Genoa, 16148 Genoa, Italy; carola.torazza@unige.it (C.T.); francesca.bacchetti@edu.unige.it (F.B.); marco.milanese@unige.it (M.M.)
[2] Scientific Institute for Cancer Research (IRCCS), Ospedale Policlinico San Martino, 16132 Genova, Italy; barbara.marengo@unige.it
[3] Department of Experimental Medicine (DIMES), University of Genova, 16132 Genoa, Italy; mario.passalacqua@unige.it (M.P.); elaheh.khaledizadeh@edu.unige.it (E.K.); stefania.vernazza@unige.it (S.V.)
[4] Centro 3R, Department of Information Engineering, University of Pisa, 56122 Pisa, Italy
* Correspondence: alfei@difar.unige.it (S.A.); cinzia.domenicotti@unige.it (C.D.); Tel.: +39-010-355-2296 (S.A.)

Section S1

*Previous Investigation with BPPB on Cancer Cells: A Brief Summary **

Cancer cells display a lot of mitochondrial dysfunctions, including change in energy metabolism, increased transmembrane potential and high production of reactive oxygen species (ROS), which can offer the opportunity to exploit them to preferentially target cancer cells, thus enhancing the therapeutic selectivity of new cancer drugs. In fact, mitochondrial dysfunction in cancer cells might render them more disposed to induce cytochrome C or apoptosis inducing factors (AIFs) release. Therefore, the use of mitochondria-targeting compounds, which can preferentially trigger the release of apoptotic factors from them, could be an attractive strategy to selectively kill cancer cells. It has been reported that molecules possessing the TPP group, due to their large cationic radius, are capable to electrostatically interact with the anionic constituents of cytoplasmic membrane of cancer cells. Once attached to cells surface, TPP-molecules can both start to damage the membrane and readily enter the cancer cell, due to their lipophilic character and target mitochondria, whose double enveloping membranes are even more negative than external cytoplasmic membrane, thus strongly attracting TPP-based compounds. It has been reported that TPP-based compounds can collect in mitochondria resulting in 100- to 500-fold higher accumulation, impairing essential functions for cell survival and causing cell death. Initially, the widespread use of TPP groups as the carrier of choice for mitochondrial targeting was based on a belief that the TPP moiety was an inert carrier. However, recent studies have established that the TPP moiety has detrimental effects on mitochondrial bioenergetics. TPP-based conjugates can increase proton leak and uncoupled mitochondrial oxidative phosphorylation (OXPHOS), thereby decreasing the efficiency of ATP generation. Additionally, mitochondria-targeting molecules may kill drug-resistant cancer cells by initiating mitochondrial outer membrane permeabilization independently of upstream signalling processes, which may be impaired in cancer cells. On this information, last year, we synthetized and characterized a *bis*-triphenyl

phosphonium (BTPP)-bola-amphiphilic (BA) molecule (BPPB), whose potent anticancer effects were first assessed against neuroblastoma (NB) cells sensitive to etoposide (ETO) (HTLA 230 cells) and the multidrug resistant counterpart (HTLA ER cells) used also in this study. Limited toxicity was instead observed against different not tumoral mammalian cell lines and red blood cells (RBCs). Subsequently, the antiproliferative effects of BPPB were assessed on cutaneous metastatic melanoma cells and preliminary experiments to confirm an oxidative toxicity as a mechanism of action were carried out by evaluating BPPB capability to induce ROS production. Potent anticancer effects strictly connected with increased ROS generation were observed against vemurafenib (PLX4032)-sensitive MeOV ($BRAF^{V600E}$) and MeTRAV ($BRAF^{V600D}$) cells, thus reinforcing the assumption of a possible oxidative toxicity, causing high levels of ROS, oxidative stress (OS), impairment of cell functions essential for life and cells death. Very recently, the effects of BPPB on cells viability were assessed also on two *in vitro* selected PLX4032 resistant CMM cell lines (PLX-R MeOV $BRAF^{V600E}$ and MeTRAV $BRAF^{V600D}$), no longer inhibited by the initially functioning PLX4032, with excellent results despite their acquired tolerance to this available therapeutic option. Non tumorigenic HaCaT human keratinocytes and red blood cells (RBCs) were used to successfully evaluate the possible clinical development of BPPB as topical agent to treat skin melanoma lesions. The existence of possible correlations between either cells viability (%) or ROS generation and time of exposure or BPPB concentrations were studied. Based on literature reports, without further investigations, we assumed that BPPB to exert the observed effects on all the tumoral cells studied, could have entered NB cells, targeted mitochondria and disrupted their membrane potential, essential to produce ATP. Relying on literature data again, we assumed that BPPB could have caused programmed death of cells by induction of apoptosis, necroptosis or autophagy. Moreover, the bola-amphiphilic structure, gifting BPPB with the capacity to self-assemble in water solution in nanosized spherical vesicles and to generate nanoaggregates, could have further increased its toxicity to mitochondria.

Section S2: Excel graphs

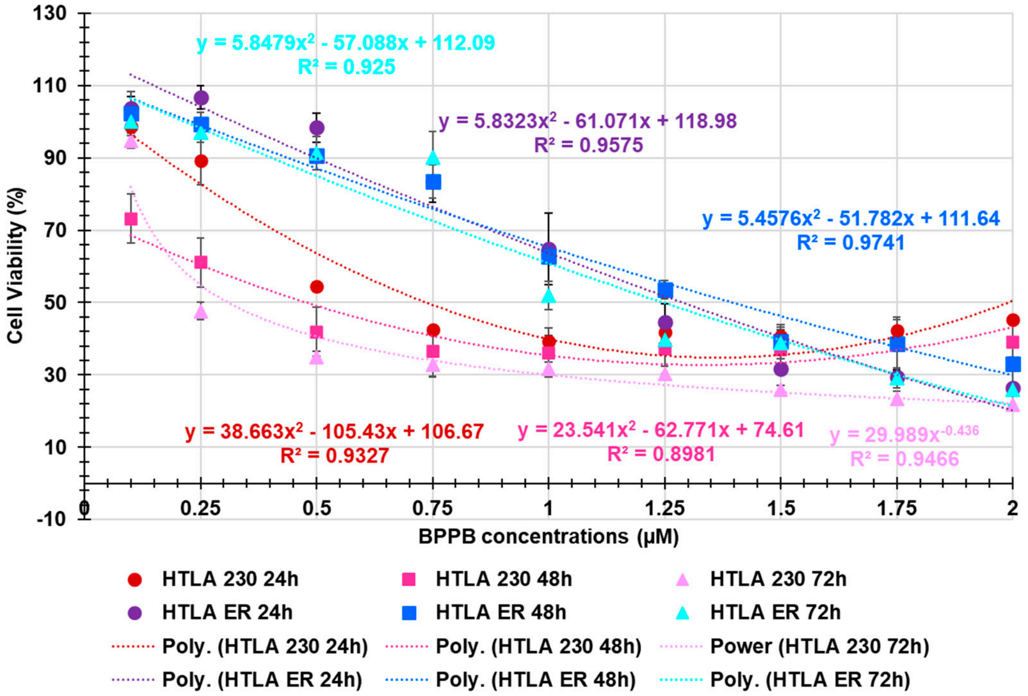

Figure S1. Dispersion graphs (no lines) of cell viability (%) of HTLA 230 and HTLA ER neuroblastoma cells vs. increasing BPPB concentrations (0.1-2.0 µM) after 24 hours (red and purple round indicators, respectively), 48 hours (pink and blue square indicators, respectively) and 72 hours (light pink and sky-blue triangular indicators, respectively) of exposure. Dotted lines in the same colours of indicators represent the nonlinear regression models which best fit the data of dispersion graphs, according to R^2. In same colours, the associated R-squared values and the equations expressing the mathematical relationship existing between data, have been shown.

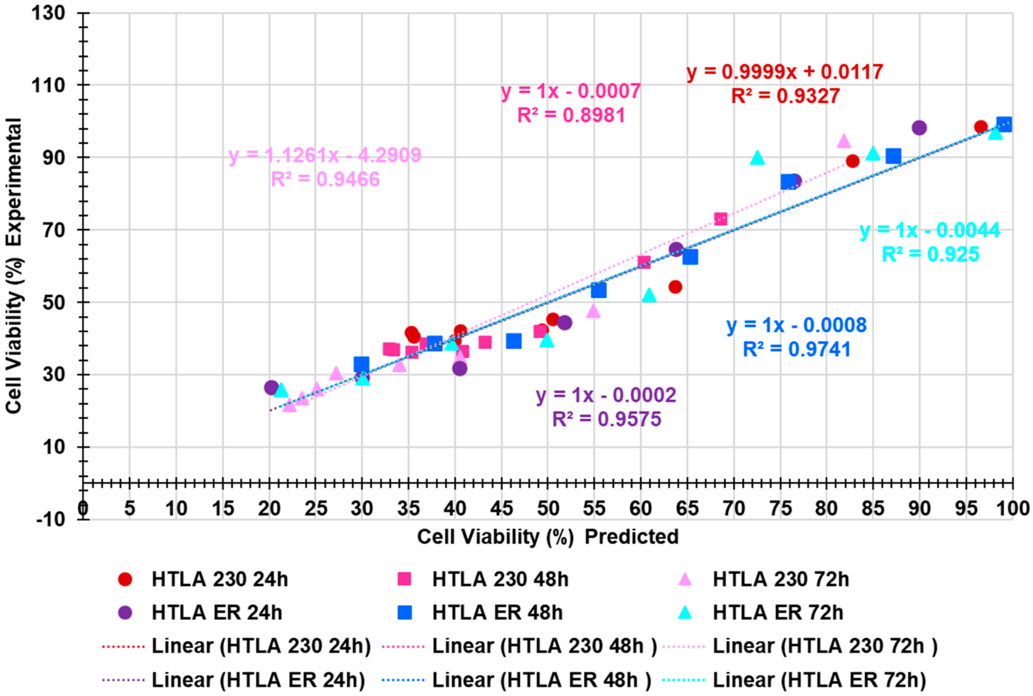

Figure S2. Dispersion graphs (no lines) of experimental data (%) of HTLA 230 and HTLA ER neuroblastoma cells vs. data predicted by the models selected based on R^2 after 24 hours (red and purple round indicators, respectively), 48 hours (pink and blue square indicators, respectively) and 72 hours (light pink and sky-blue triangular indicators, respectively) of exposure. Dotted lines in

the same colours of indicators represent the related linear regression models. In same colours, the associated R^2 values and the equations expressing the mathematical relationship existing between data, have been shown.

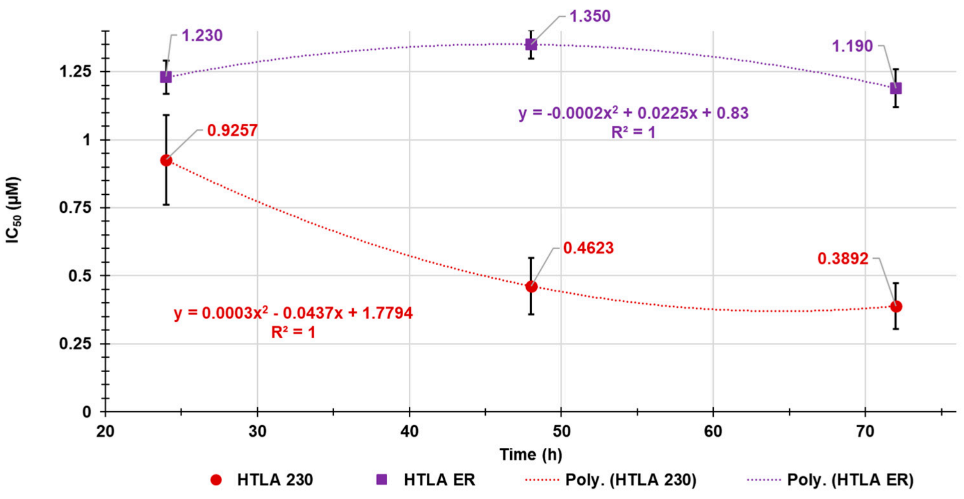

Figure S3. Dispersion graphs of IC_{50} values of BPPB towards HTLA 230 (red round indicators) and HTLA ER (purple square indicators) as functions of exposure timing with related second order polynomial regression models (punctuated red and purple lines), their equations and R^2 values, as provided by Microsoft Excel 365 software.

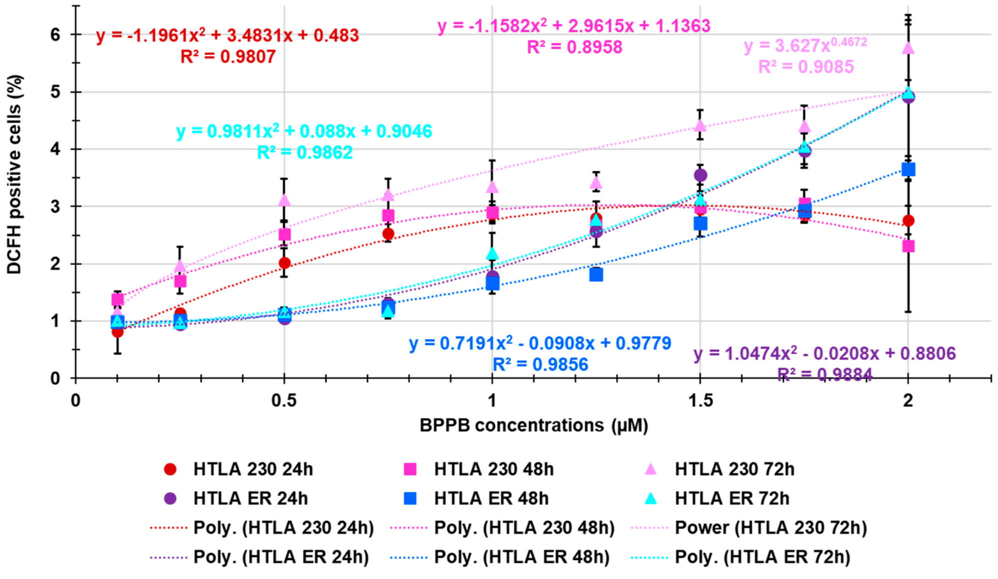

Figure S4. Dispersion graphs (no lines) of DCFH positive cells (%) of HTLA 230 and HTLA ER neuroblastoma cells vs. increasing BPPB concentrations (0.1-2.0 μM) after 24 hours (red and purple round indicators, respectively), 48 hours (pink and blue square indicators, respectively) and 72 hours (light oink and sky-blue triangular indicators, respectively) of exposure. Dotted lines in the same colours of indicators represent the nonlinear regression models which best fitted the data of dispersion graphs, according to R^2. In same colours, the associated R-squared values and the equations expressing the mathematical relationship existing between data, have been shown.

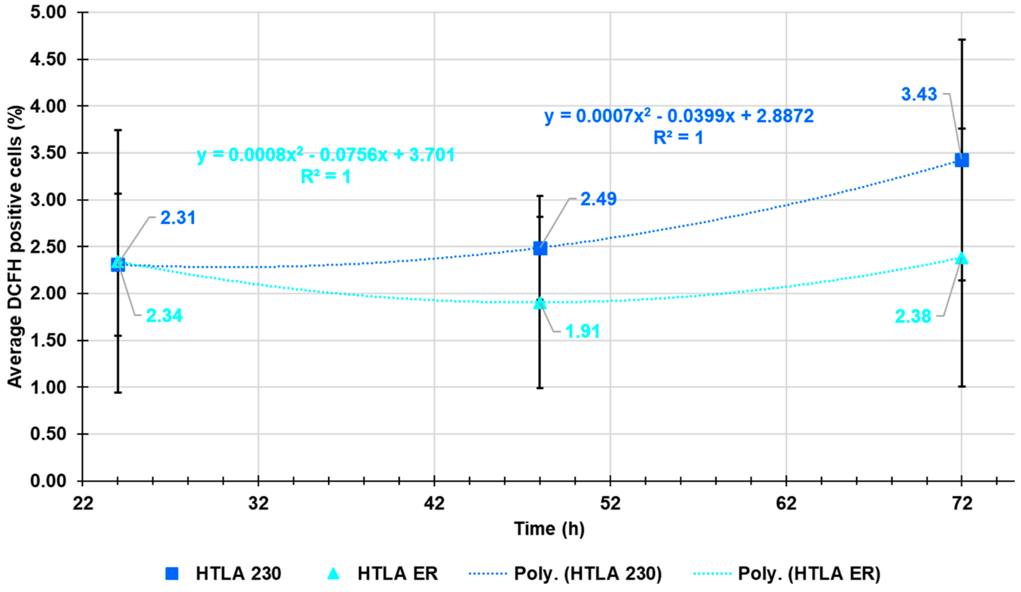

Figure S5. Dispersion graphs of the average DCFH positive cells (%) observed for both cells' lines vs. exposure timing, with the related nonlinear regression models which best fitted the data and their R^2 values.

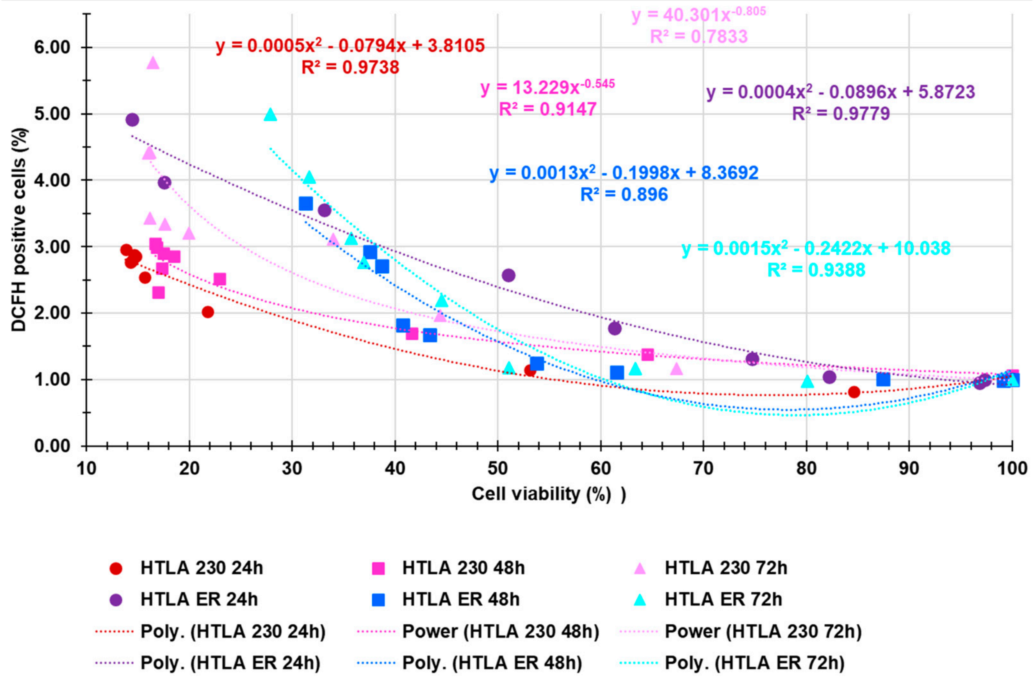

Figure S6. Dispersion graphs of the DCFH positive cells (%) vs. cell viability (%) (round red and purple, square pink and blue and triangular light-pink and sky-blue indicators without lines) observed at the same concentrations of BPPB, for both HTLA 230 and HTLA ER cells, respectively, after 24, 48 and 72 of treatment. Best fitting nonlinear regression models (dotted lines), their equations and the related R^2 values.

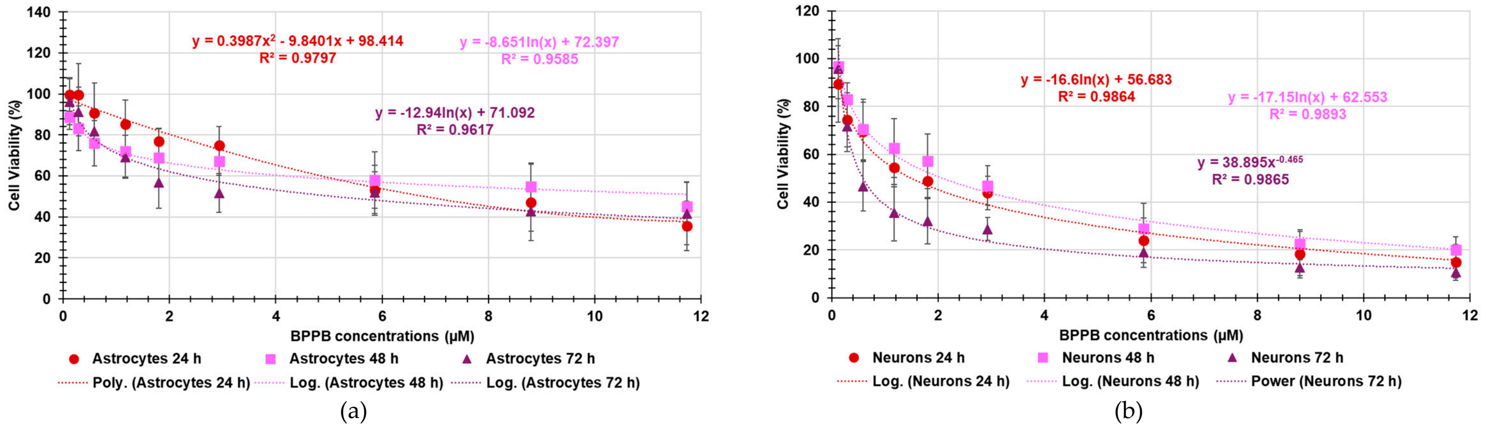

Figure S7. Dispersion graphs (no lines) of cell viability (%) of primary spinal cord astrocytes (a) and cortical neurons (b) vs. increasing BPPB concentrations (1.1-11.7 μM) after 24 hours (red round indicators), 48 hours (pink square indicators) and 72 hours (purple triangular indicators) of exposure. Dotted lines in the same colours of indicators represent the nonlinear regression models which best fitted the data of dispersion graphs, according to R^2. In same colours, the associated R-squared values and the equations expressing the mathematical relationship existing between data, have been shown.

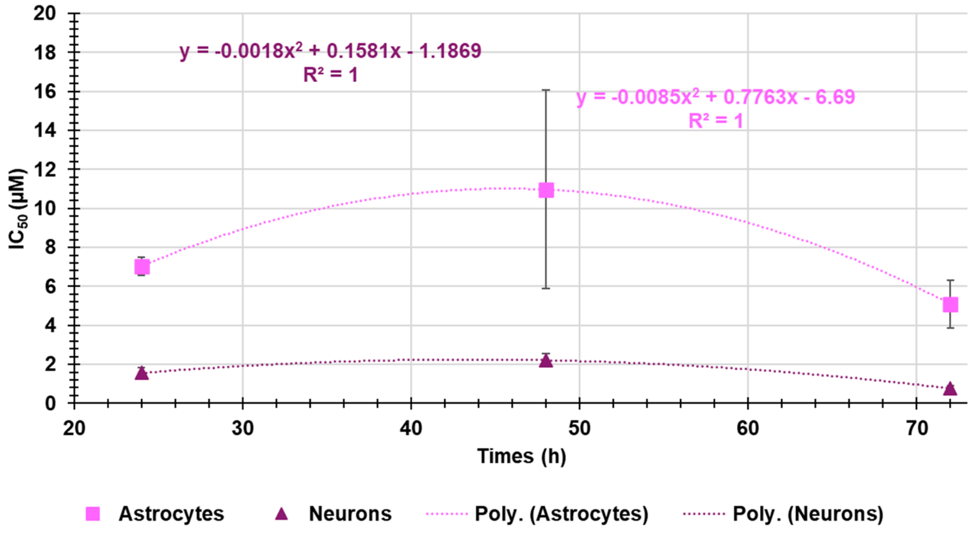

Figure S8. Dispersion graphs of the average DCFH positive cells (%) observed for both astrocytes and neurons vs. exposure timing, with the related nonlinear regression models which best fitted the data and their R^2 values.
